# The warfare for plant highway: vascular plant–microbe interaction pinpoints lignin

**DOI:** 10.1007/s44154-022-00047-0

**Published:** 2022-06-23

**Authors:** Gan Ai, Dong-Lei Yang, Daolong Dou

**Affiliations:** 1 College of Plant Protection, Nanjing Agricultural University, Nanjing, 210095 China; 2 State Key Laboratory of Crop Genetics and Germplasm Enhancement, Nanjing Agricultural University, Nanjing, 210095 China

**Keywords:** Phloem pathogens, Xylem pathogens, Vascular resistance, MAPK cascade, Lignin

## Abstract

Plant vascular pathogens are one kind of destructive pathogens in agricultural production. However, mechanisms behind the vascular pathogen-recognition and the subsequent defense responses of plants are not well known. A recent pioneering study on plant vascular immunity discovered a conserved MKP1-MPK-MYB signaling cascade that activates lignin biosynthesis in vascular tissues to confer vascular resistance in both monocot rice and the dicot Arabidopsis. The breakthrough provides a novel view on plant immunity to vascular pathogens and offers a potential strategy for the future breeding of disease-resistant crops.

## Main text

The plant vascular system is a complicated network of connecting and conducting tissues that are mainly composed of xylem and phloem elements. The xylem transports water and solutes from the soil upwards, while the phloem transports nutrients and signaling molecules throughout the plant body in multiple directions. Thus, the vascular system acts like a highway and is essential for vascular plants to survive and thrive by the delivery of resources and maintenance of long-distance communication (Agusti and Blazquez, 2020; Lucas et al., 2013).

**Fig. 1:**
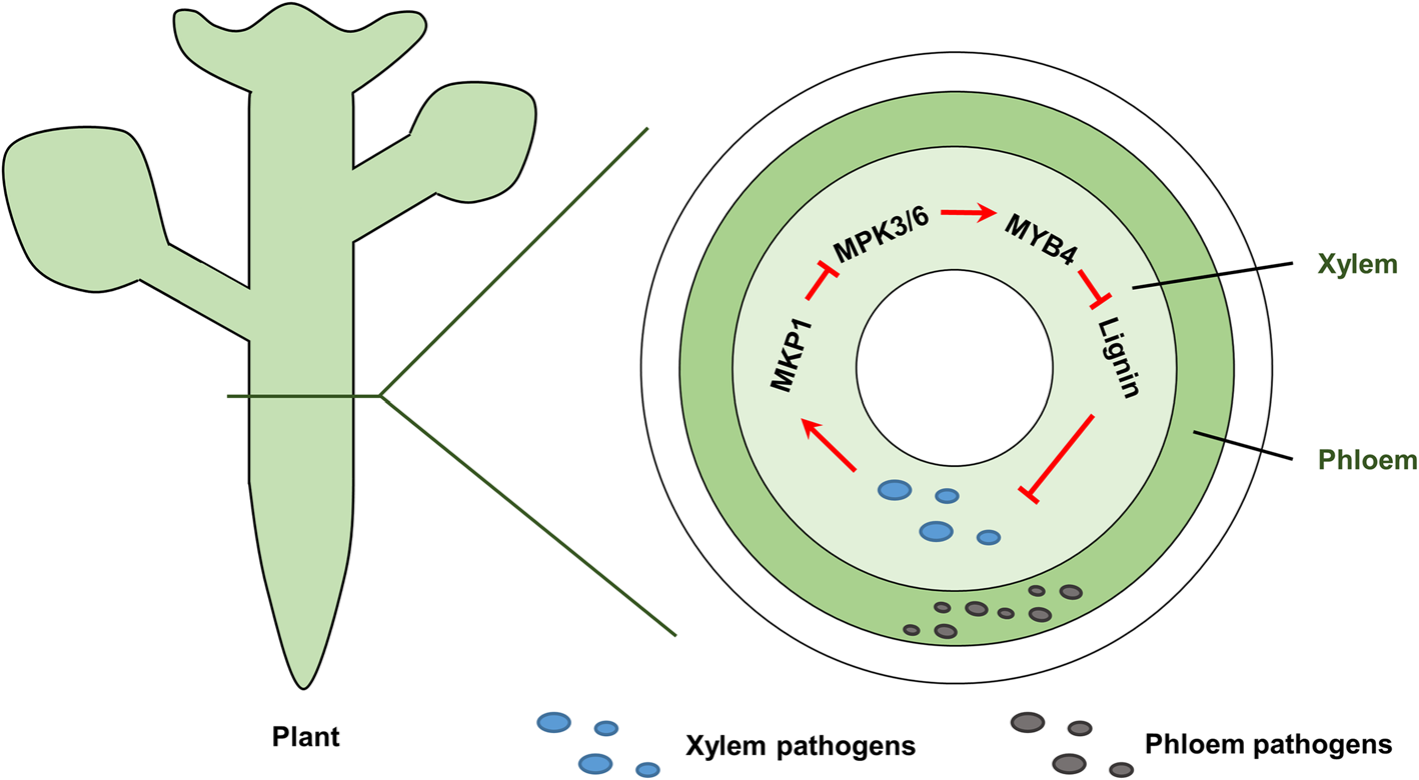
A illustration for the function of MKP1-MPK3/6-MYB cascades in plant vascular resistance discovered by Lin et al. (Lin et al., 2022). Xylem pathogens and phloem pathogens are vascular pathogens proliferating in vascular system. In xylem, infection of vascular pathogens inhibits MAPK phosphorylation pathway by inducing the *MKP1* gene expression, resulting in inactivation of the MYB transcription factors that negatively regulate lignin biosynthesis genes expression

Enormous fungal, bacterial, and oomycete pathogens can infect, colonize and proliferate in the plant vascular system to cause many destructive diseases that can wipe out entire plants (Bendix and Lewis, 2018; Yadeta and Thomma, 2013). Since the phloem is characterized by living cells with a high osmotic pressure that makes penetration difficult, only certain pathogens, including phytoplasmas, spiroplasmas and viruses, may access and live in this sequestered and protected environment. They are called phloem pathogens or phloem-limited pathogens (Bendix and Lewis, 2018; Lewis et al., 2022) (Fig. 1). One notorious example is Citrus greening or Huanglongbin caused by *Candidatus* Liberibacter asiaticus (*C*Las). *C*Las appears to be adapted to the phloem environment by distinct strategies. For example, its genome contains multiple components necessary for aerobic respiration. No known resistant, only some tolerant, citrus varieties are available, and phloem-specific defense responses remain poorly characterized so far (da Graca et al., 2016).

More microorganisms proliferate in the xylem to cause vascular wilt diseases, which are called xylem-invading pathogens or vascular wilt pathogens (Yadeta and Thomma, 2013) (Fig. 1). It has been reported that they comprise four fungal genera (*Ceratocystis*, *Ophiostoma*, *Verticillium* and *Fusarium*), seven bacterial genera (*Clavibacter*, *Curtobacterium*, *Erwinia*, *Pantoea*, *Ralstonia*, *Xanthomonas* and *Xylella*) and one oomycete genus (*Pythium*). Vascular wilt pathogens are generally soil-borne and enter their host xylem vessels via different routes. To satisfy their nutritional requirements in the xylem, the pathogens can produce enzymes and/or toxins to digest host cell walls, invade neighboring cells, or induce nutrient leakage from surrounding tissues. As a consequence, the xylem system is destroyed or obstructed, and the leaves wilt and die, which may ultimately lead to impairment and even death of the whole plant. The diseases are usually difficult to control because many vascular pathogens are soil-borne and hard to eliminate. One potential strategy is introducing double strand RNA (dsRNA) or hairpin RNA (hpRNA) targeting the essential genes of pathogens into host plants, which is also called host-induced gene silencing (HIGS). Small RNAs are transported via vascular channels from the source to various sink tissues (Ham and Lucas, 2017), indicating that host-derived small RNAs could be taken up by pathogens in spatial. Indeed, a recent study showed that highly effective trans-kingdom silencing of pathogen targets took place inside vascular through the HIGS (Zhang et al., 2022). Another well-accepted way to control vascular diseases is using genetic resistance in host plants. However, resources are very scarce (Yadeta and Thomma, 2013).

Despite the significant importance of and huge losses caused by plant vascular pathogens, studies on the plant perception of them and subsequent defense responses are obviously lagged. Some extracellular and intracellular plant receptors have been described to mediate defense against vascular wilt pathogens. For example, rice Xa21, a leucine-rich repeat (LRR) receptor-like kinase (RLK), may recognize RaxX (required for activation of XA21-mediated immunity X), a ligand from *Xanthomonas oryzae* pv. *oryzae* (*Xoo*), to mediate resistance against *Xoo* (Ercoli et al., 2022). Tomato Ve1 is a LRR receptor-like protein (RLP) that confers resistance against race1 isolates of *Verticillium dahliae* and *V.albo-atrum* by perceiving Ave1 effectors, whose homologs are widely distributed in several vascular wilt fungus pathogens (de Jonge et al., 2012). Tomato I-2 is a cytoplasmic coiled-coil–nucleotide-binding site–LRR receptor protein that recognizes the effector protein Avr2 (*SIX3*) from *Fusarium oxysporum* f. sp. *Lycopersici* (Ma et al., 2013). Due to the interaction deep in the plant interior and lack of resistant germplasm, plant vascular immunity and the interaction between vascular wilt pathogens and their hosts remains largely obscure.

Recently, Prof. Zuhua He at Chinese Academy of Sciences and his colleagues conducted a pioneering study on plant vascular immunity, and discovered a conserved MKP1-MPK-MYB signaling cascade that activates lignin biosynthesis in vascular tissues to confer vascular resistance in both monocot rice and the dicot Arabidopsis (Lin et al., 2022). They first utilized Arabidopsis to conduct a genetic screen for nonhost resistance to *Xoo* (*ntx*) mutants. Interestingly, one mutant, *ntx1,* appears to be more susceptible to the nonadapted pathogen *Xoo* and the adapted vascular pathogen *Xanthomonas campestris* pv. *campestris* (*Xcc*), but shows enhanced basal resistance to *Pseudomonas syringae* pv. *tomato* (*Pst* DC3000), a leaf mesophyll–specific bacterial pathogen, suggesting that *NTX1* is specifically involved in vascular immunity. They cloned the responsible gene for *ntx1* and found it encoding MKP1, a mitogen-activated protein (MAP) kinase phosphatase (Lin et al., 2022).

The MKP1 was found to deactivate MPK3 and MPK6 via direct interaction and dephosphorylation, which is consistent with previous results (Ulm et al., 2002). Specially, *Xoo* infection inhibits MPK3 phosphorylation, which is dependent on MKP1, suggesting that vascular resistance is ensured by MPK1-mediated dephosphorylation of MPK3. Furthermore, the authors showed that almost all genes of the lignin biosynthesis pathway were down-regulated and the lignin accumulation level was reduced in the *ntx1* mutant during *Xoo* infection. Since MYB4 is a vein-specific expressed transcriptional factor and negatively regulates lignin biosynthesis (Hemm et al., 2001), the authors presented several lines of evidence to prove that lignin biosynthesis is dynamically controlled by MPK3-mediated phosphorylation of MYB4. Similarly, in the *Xoo* native host rice, a parallel signaling cascade is conserved, by which, an OsMKP1-OsMPK6 protein phosphorylation cascade regulates OsMYB102/OsMYB108-mediated lignin biosynthesis in vascular defense. Collectively, the authors disclose that an MKP1-MPK3-MYB signaling cascade of Arabidopsis and rice controls lignin-based vascular immunity. Pathogen infection induces the *MKP1* gene expression and thus attenuates the MAPK phosphorylation pathway, leading to inactivation of the MYB transcription factors that negatively regulate lignin biosynthesis genes (Fig. 1).

It is of great interest that the MKP1-MAPK cascade plays an opposite role against nonvascular pathogens (Lin et al., 2022). MAPK cascades have been well studied in plants, especially in signaling plant defense against pathogen attack. MAPKs are activated when plants sense pathogen MAMPs or effectors and then induce multiple defense responses through phosphorylation of target proteins (Meng and Zhang, 2013), including MYB4*.* Phosphorylation of MYB4 inhibits lignin biosynthesis. Since the *MYB4* gene is specifically expressed in the vascular bundle, lignin deposition in the xylem is fine-tuned by the MKP1-MPK3/6-MYB phosphorylation cascade during plant vascular pathogen infection to confer resistance (Lin et al., 2022). The different roles of the MKP1-MAPK cascade against nonvascular and vascular pathogens may provide molecular targets and ideas for future disease resistance breeding.

Lignin is a phenolic polymer mainly deposited in the secondary cell wall. It not only provides mechanical strength and imperviousness to cell walls for plant vascular system development and functions, but also has multiple roles in plant defenses (Yoon et al., 2015). It has been reported that Xa21-mediated *Xoo* resistance is related to lignin accumulation (Shamsunnaher et al., 2020), suggesting that lignin-mediated vascular defense functions in race-specific resistance. This study (Lin et al., 2022) not only pinpoints lignin as the mechanical barrier against vascular pathogens, but also suggests the possible principal virulent mechanisms specific to vascular pathogens, as well as indicates the future development of crop resistance strategies.

## Data Availability

Not applicable.

## Abbreviations

T3SS: Type III secretion system
MAMPs: Microbe-associated molecular patterns
LRR: Leucine-rich repeat
RLK: Receptor-like kinase
RLP: Receptor-like protein
MAP: Mitogen-activated protein
